# Fistula in Perineo

**Published:** 1886-11

**Authors:** M. B. Hutchins

**Affiliations:** Lawrenceville, Ga.


### FISTULA IN PERINEO.

BY M. B. HUTCHINS, M. D., LAWRENCEVILLE, GA.

W. T., aged 36, carpenter, married. About June 1, 1885, a furuncle (patient describes as a "rising") appeared in superficial perineal tissue, about one and one-half inches anterior to the anus, to the right of median *raphe*. In eight or nine days "pointed" and discharged its contents—small in quantity. The opening then closed and the case seemed well. In about two days, however, the place again became swollen and painful and pus was again discharged. This routine of symptoms continued until August, 1886, when I first saw the case.

August 11, the case appeared as follows: An induration, not elevated, about the size of a small pecan, extended in the direction of the sphincter ani. At the anterior extremity of this formation the skin was raised in a manner best described as resembling half of a large bean hollowed out, convexity upward. About the center was an opening only large enough to allow the entrance of an ordinary *pin*. This opening was enlarged to the size of the elevation described.

Nothing further was done until August 16. A probe was then passed into the opening, entering a canal which followed the induration above described to a depth of ⅞ of an inch. There was found to be no anal or urethral connection. Patient objected to the operation of destroying with the knife, so treatment was as follows: The external opening was enlarged. A small blunt probe was then dipped in undilute carbolic acid, then dipped in water, diluting acid a little. Probe was then introduced applying the acid over the whole internal surface. The flat end of probe was then introduced and twisted around in order to keep mouth of canal open. The canal was not larger than an ordinary match.

Canal had closed, August 18, from bottom, leaving only a depth of ½ inch. August 20, only about ⅓ inch in depth. Patient did not return until 23d, when the external opening was found completely closed, but the fistula had given little pain. Upon opening found only a drop or two of pus. Again patient remained away until mouth of fistula had closed. Again cut on August 28. The canal was now about ¼ inch in depth and there was no pus.

Treatment was kept up under these difficulties until September 22, the canal healing very slowly. Fistula then about ⅛ inch in depth and the induration correspondingly lessened. Surrounding the opening were now noticed three or four shallow excavations one-sixth to one-third inch in depth. All these were destroyed by the knife, save the smallest. Two days later fistula was healing from the bottom and the fistula was "filled" on a level with remaining small excavation. September 29 this was opened and all corners trimmed away. Patient discharged as cured October 1.

I neglected to mention that, being impracticable to keep mouth of fistula open with "tent," the patient was given a probe to use for this purpose, but failed to do so.

No syphilitic history or cachexia.

---

FALSE HONORS—"TAIT'S OPERATION" NO MORE, BUT "BATTEY'S OPERATION."—Like the operation of ovariotomy, America is slowly but surely receiving the acknowledgment due her surgeons for the early pioneer work in abdominal surgery in diseases of women. The reaction has set in, and Dr. Robert Battey, of Rome, Georgia, is now accorded the honor of introducing to the profession the operation that has in it certain principles which, if not abused in our enthusiasm, will mark one of the greatest epochs in the special field of surgery known as gynæcology. While the profession have much to admire in Mr. Lawson Tait, we must not forget to "give honor to whom honor is due." —*Homœopathic Journal of Obstet.*

